# Turner syndrome and neuropsychological abnormalities: a review and case series

**DOI:** 10.1590/1984-0462/2025/43/2023199

**Published:** 2024-09-09

**Authors:** Bruna Baierle Guaraná, Marcela Rodrigues Nunes, Victória Feitosa Muniz, Bruna Lixinski Diniz, Maurício Rouvel Nunes, Ana Kalise Böttcher, Rafael Fabiano Machado Rosa, Rafaella Mergener, Paulo Ricardo Gazzola Zen

**Affiliations:** a Universidade Federal de Ciências da Saúde de Porto Alegre, Porto Alegre, RS, Brazil.; b Irmandade da Santa Casa de Misericórdia de Porto Alegre, Porto Alegre, RS, Brazil.

**Keywords:** Turner syndrome, Neurodevelopmental delay, Intellectual disability, Learning disabilities, Monosomy X, Síndrome de Turner, Atraso do neurodesenvolvimento, Deficiência intelectual, Dificuldade de aprendizagem, Monossomia X

## Abstract

**Objective:**

The objective of this study was to establish the genotype-phenotype correlation between karyotype results and the neurological and psychiatric alterations presented in patients with Turner syndrome (TS).

**Methods:**

A retrospective study was conducted on the medical records of 10/140 patients with TS and neurophysiological abnormalities seen at a university hospital in southern Brazil. In addition, a literature review spanning the period from January 1, 2012 to January 1, 2023 was carried out using the PubMed and Virtual Health Library databases.

**Results:**

Our study showed a potential correlation between neurological and psychiatric alterations in patients with TS. These findings are in accordance with those described in literature such as a high prevalence of learning or intellectual disabilities. However, our sample found more seizure episodes than those reported in other studies.

**Conclusions:**

The correlation established could be due to X chromosome dose–effect, as the review suggests that sex chromosome number and hormonal development can be associated with verbal, social, and cognitive skills or impairments.

## RESUMO

**Objetivo:**

O objetivo deste estudo foi realizar a correlação genótipo-fenótipo entre os resultados de cariótipos e as alterações neurológicas e psiquiátricas apresentadas em pacientes com síndrome de Turner (ST).

**Métodos:**

Um estudo retrospectivo foi conduzido nos registros médicos de 10/140 pacientes com ST e deficiências neuropsicológicas atendidos pelo Serviço de Genética Clínica de um hospital do sul do Brasil. Ademais, uma revisão foi realizada pelas plataformas científicas PubMed e Biblioteca Virtual em Saúde (BVS) de artigos publicados entre 1^o^ de janeiro de 2012 e 1^o^ de janeiro de 2023. Foram selecionados 14 artigos em uma análise em duas etapas.

**Resultados:**

Nossa pesquisa mostrou potencial correlação entre alterações neurológicas e psiquiátricas em pacientes com ST. Esses achados corroboram os descritos por outros autores — como a elevada prevalência de dificuldades de aprendizagem ou intelectuais. Entretanto, nossa amostra encontrou mais episódios convulsivos do que os descritos em outros estudos.

**Conclusões:**

A correlação estabelecida pode ser devida ao efeito dose-dependente do cromossomo X, visto que nossa revisão sugere que o número de cromossomos sexuais ou o desenvolvimento hormonal está associado a habilidades ou deficiências verbais, sociais e cognitivas.

## INTRODUCTION

Turner syndrome (TS), also referred to as congenital ovarian hypoplasia syndrome, was first described by Henri Turner and is characterized by the complete or partial deletion, or nonfunctioning, of one X chromosome in women.^1,2^ The cytogenetic causes of the syndrome are diverse. While half of the TS population has complete monosomy (45,X), some may display isochromosome Xq, ring chromosomes, and Xp or Xq deletion.^3^ Even Y chromosome mosaicism may be found.

TS is the most common female sex chromosome aneuploidy (SCA), and it has an incidence of 1 in 2,000 live births.^4,5^ However, we highlight that these data may present a bias due to the underdiagnosis or the delayed diagnosis of the syndrome in girls with a mild phenotype.^6,7^ The main clinical features of TS encompass short stature, caused by a deletion or inactivation of the short stature homeobox (*SHOX*) gene (Xp22.33), and congenital ovarian hypoplasia, but cardiac malformations, decreased auditory acuity, and renal abnormalities are frequent.^8,9^ Furthermore, patients can develop neurological and psychiatric disorders, such as seizures, shyness, learning difficulties, and cognitive impairment.^8^

The syndrome can be traced prenatally with abnormal obstetric ultrasound findings, such as nuchal cystic hygroma, increased nuchal translucency, cardiac malformations, poly- or oligohydramnios, and nonimmune fetal hydrops. It can also be identified in newborn females presenting lymphedema of the hands and feet, webbed neck, nail dysplasia, narrow and high-arched palate, and short fourth metacarpals or metatarsals. Other traits include spaced nipples, low hairline at the neck, and cubitus valgus.^8^

It is established that SCAs are associated with neuropsychological disabilities, which include neurodevelopmental, neurological disorders, or behavioral alterations. Other examples of sex chromosome disorders are the Klinefelter syndrome (KS) (47,XXY), the 47,XYY syndrome, and trisomies or even tetrasomies of the X chromosome. However, the only monosomy compatible with human life is TS.^7,10^

Neurodevelopmental disorders, such as autism spectrum disorder (ASD) and attention deficit hyperactivity disorder (ADHD), are associated with structural and functional changes in neural circuit, including the X chromosome.^11-16^ But TS is known to present specific neurocognitive deficits (e.g., problems with visuospatial organization, general and specific learning disabilities, mostly with calculations, attention, and memory).^8,17^ Additionally, patients are described with social anxiety, severe shyness, low self-esteem, and reduced social interaction, perhaps correlated with impaired development of secondary sexual characters.^18^ We will discuss the main neurological changes and cognitive impairments throughout this article.

This article aims to contribute to the existing body of knowledge by conducting a case-series analysis of TS patients with neuropsychological abnormalities within the context of the Clinical Genetics Hospital Service in the south of Brazil. The study seeks to establish a genotype-phenotype correlation by examining the clinical and cytogenetic findings of the studied patients combined with a literature review in article databases with scientific database platforms.

## METHOD

A total of 140 female patients with TS were seen by the UFCSPA Clinical Genetics Service at San Antonio Children’s Hospital (HCSA) in southern Brazil, from 1975 to 2019. The medical records of these patients were screened for phenotypic alterations and neurological conditions, such as epilepsy and neurodevelopmental disorders, including intellectual disabilities and global developmental delay. From this pool, 10 individuals (7%) with neurophysiological abnormalities were selected as the study sample. The case study performed is retrospective through the analysis of medical records in order to correlate genotype-phenotype. However, given the broad time range covered by the examined records, there is a lack of standardization of neurodevelopmental disorders diagnosis.

A literature review was conducted to gather information on TS, delayed neuropsychomotor development, and intellectual disability through PubMed (Medline) and Virtual Health Library (Lilacs/Medline) for articles published between January 1, 2012 and January 1, 2023. The articles were indexed using DeCs and Mesh descriptors, and the exact search terms used were: ((“turner syndrome”) OR (“turner syndrome”[MeSH Terms])) AND ((“intellectual disability”) OR (“intellectual disability”[MeSH Terms])); review and systematic review; and ((intellectual disability[MeSH Terms]) OR (neurodevelopmental disorders[MeSH Terms])) AND (turner syndrome[MeSH Terms], in which 16 articles were selected. On the BVS platform, the terms used were (turner syndrome) AND (developmental disorders), and (turner syndrome) AND (intellectual disability), in which 13 articles were selected.

Articles were selected in a two-step analysis: title and abstract screening followed by a full-text read. The inclusion criteria were original articles or literature reviews, with or without a case report description, written about TS and delayed neuropsychomotor development or intellectual disability. Articles written in English, Spanish, and French were also included. It excluded articles that did not meet the inclusion criteria, articles that were not found with full text, and repeated articles-both in PubMed and BVS platforms. Finally, a total of 14 articles were selected from PubMed and BVS scientific platforms.

## RESULTS

**Figure 1:**
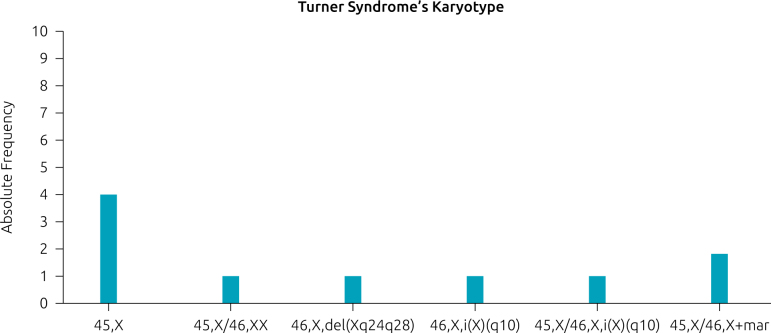
Frequency of different karyotypes in patients with Turner syndrome neuropsychological abnormalities population at the Clinical Genetics Hospital Service in the south of Brazil.

Among the sample, the distribution of karyotype was as follows: 45,X (40%), 45,X/46,X+mar (20%), 46,X,del(Xq24q28) (10%), 46,i(X)(q10) (10%), 45,X/46,X,i(X)(q10) (10%), 45,X/46,XX (10%). In Figure 1, it is possible to identify cytogenetic findings.

**Figure 2:**
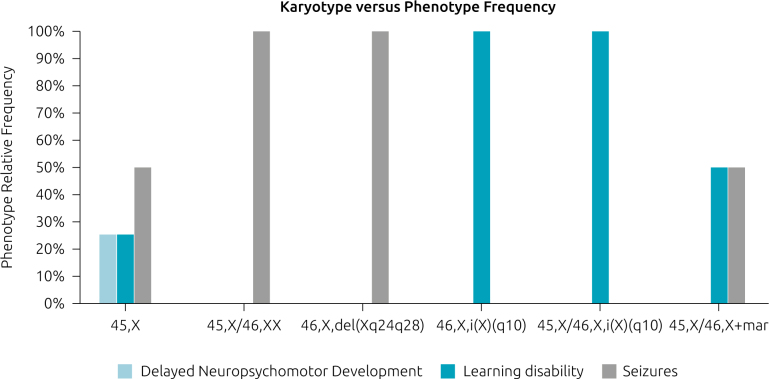
Frequency of clinical findings and karyotype prevalence in patients with Turner syndrome and neuropsychological abnormalities at the Clinical Genetics Hospital Service in the south of Brazil.

Our sample showed different neuropsychological abnormalities, as specified in Figure 2. Half of the sample (50%) showed seizures-one or more episodes. Almost half (40%) showed learning disabilities, which were mostly intellectual disabilities and cognitive impairments. Only one case (10%) had delayed neuropsychomotor development.

As shown in Figure 1, our sample showed a heterogeneous chromosomal constitution. The most frequent karyotype was X monosomy (45,X) in 40% of cases. The second with higher frequency was mosaicism with a marker chromosome (45,X/46,X+mar) in 20% of cases. Regarding clinical findings, the most common neuropsychological abnormalities were seizures-either one or more episodes. In Tables 1 and 2, we can also identify the most prevalent neuropsychiatric findings, such as those in our own sample and those in other literature review studies. The age of diagnosis is presented in Table 1.

**Table 1:** Original sample review of genotype-phenotype correlation in patients with Turner syndrome and neuropsychological findings.

	Patient	Age	Main features	Karyotype
Our sample	P1	2 y	Seizures	45,X
	P2	11 m	Seizures	45,X
	P3	24 y	Learning/cognitive disability	45,X
	P4	14 y	Delayed neuropsychomotor development	45,X
	P5	18 y	Seizures	45,X/46,XX
	P6	9 y	Seizures	45,X/46,X+mar
	P7	9 y	Learning/cognitive disability	45,X/46,X+mar
	P8	16 y	Learning/cognitive disability	45,X/46,X,i(X)(q10)
	P9	18 y	Learning/cognitive disability	46,X,i(X)(q10)
	P10	34 y	Seizures Acute facial palsy Depression	46,X, del(Xq24q28)

P: patient; y: years; m: months.

**Table 2:** Literature review of genotype-phenotype correlation in patients with TS and neuropsychological findings.

Study	Patient	Age	Main features	Karyotype
Hu et al.^19^	P1	10 y	Learning/cognitive disability Skin pigmentation	mos 46,X,r(X)p22q32)[0.60]/45,X[0.40]
	P2	13 y	Learning/cognitive disability Delayed neuropsychomotor development	mos 46,XX[20]/46,r(X)(p22q22)[80]
	P3	13 y	Learning/cognitive disability Kabuki make-up syndrome Dysmorphic facial features Skeletal abnormalities Unusual dermatologic patterns	mos 45,X/46,X,r(X)
Kostopoulou et al.^20^	P1	1 y	ASD	45,X,15/46,X,i(X)
	P2	1 d	ADHD	45,X
	P3	4 m	Seizures, Hydrocephalus Spina bifida Myelomeningocele Vit B12 deficiency	46,X,r(X)(p11q13)[22]/45,X[8]
	P4	1 d	Hydrocephalus Meningitis-septicemia Cerebral necrosis	46X, deletion (X)(p11.2)
Jhang et al.^21^	P1	49 y	Learning/cognitive disability Seizures Ventricular asymmetry White matter hyperintensity	45,X[2]/47,XXX[1]/46,XX[27]
Santana Hernández et al.^22^	P1	22 y	Mild learning/cognitive disability	46,XX/45,X

P: patient; y: years; m: months; d: days; ASD: autism spectrum disorder; ADHD: attention deficit hyperactivity disorder; vit: vitamine.

## DISCUSSION

The findings of our research corroborate those found by other authors — analyzed on Table 2 ^19-22^ — showing a high prevalence of learning or intellectual disabilities (40%). However, our sample found more seizure episodes than those described in Table 2, but we must also consider that not all seizure episodes may have been reported in those studies. This bias may explain this low frequency. Besides, one of the reviewed articles studied only TS patients with mosaicism.^19^

Patients with SCAs are known to have learning disability, such as cognitive impairment. Verbal skills are usually conserved or even improved in TS, while XXY, XYY, and XXX trisomies show difficulty in these skills. Our review suggests that maybe sex chromosome number or hormonal development can be associated with verbal skills or impairments, leading to a dose-dependent effect. On the contrary, visuospatial skills are preserved in XXY and XYY trisomies, but reduced in TS and XXX. So, perhaps this preservation of visuospatial abilities could be explained by a Y chromosome or protection from male hormonal development.^10^ Executive skills are reduced in both TS and KS, so the dose-dependent effect does not explain this outcome.^23^

X-linked syndromes have an increased risk of intellectual disability (ID) and correspond to 5–10% of every ID case.^24,25^ Development disorders such as ASD and ADHD are described in literature having an association with X chromosome and its neural circuit.^11-16^ Therefore, there is a high incidence of autistic behavior in X-linked syndromes, such as Rett and X-fragile syndromes.^26-28^

The known mathematical learning disability present in TS is also described in X-fragile syndrome. While TS patients show a lack of speed in solving problems, the X-fragile patients could actually have difficulty in solving them. However, more research is needed on this topic.^29^

Social cognitive impairments are commonly found in SCAs, autosomal microdeletion, and microduplication syndromes. It can lead to functional impairment of society patient’s lives.^30^ ID is directly related to quality of life, so this may imply adaptive difficulties and social isolation, which can lead to depression, anxiety, and other psychiatric disorders.^31^ Despite this possible cause–effect relationship between X chromosome disorders and neuropsychological changes, we should also consider the environment to which the patient was exposed and the action of autosomal genes in neurodevelopment.^7^

Patients with TS have different learning disabilities, such as dyscalculia, executive disorders, and visuospatial deficits, which usually appear from the beginning of school age to adolescence.^18,32,33^ However, they usually have no difficulty in recognizing facial expressions — except for fear, which could be explained by neural circuit deficits of the encephalic tonsils — neither with verbal skills nor with reading skills.^18,34^ Actually, these verbal skills can be improved in TS.^7,35,36^ Nonetheless, because TS girls have more social anxiety, they also have more difficulty in communicating and expressing themselves.

Specific difficulties in mathematics are present in this chromosomal disorder. Our literature review showed that TS mathematical performance has a speed deficit in solving math problems, as a quick answer is required. However, performance on questions that do not require explicit calculation is equivalent to that of neurotypical patients of the same age.^17^ Also, counting and coding are usually preserved.^37,38^ Thus, studies suggest TS patients do not have difficulty in solving problems, but need more time to complete the activity.^39^ On the contrary, there are studies that really show patients having difficulty in performing addition, subtraction, and multiplication calculations.^37^ Therefore, further research is necessary to better clarify this particular disability.

TS patients also have an increased risk of developing schizophrenia and related disorders.^40^ According to Emerson and Hatton, patients with intellectual difficulties have a higher risk of developing psychiatric disorders.^31^ This result corroborates previous studies carried out by other researchers.^41-43^ From this perspective, children with these disorders receive fewer job opportunities, live in a noninclusive society and, consequently, suffer from impaired quality of life.^44^

The prevalence of depression and anxiety disorders is increased in women with TS, and they are often described as shy and socially anxious. Many factors may contribute to this, such as the lack of secondary sexual characteristics that typically play a role in heightened social pressures during adolescence, as social motivation remains unaffected. Because of the sexual development delay, patients with TS may also present low self-esteem and are at risk for developing eating disorders.^18,45^

Additionally, these conditions could also be correlated with hormone deficiencies or hormonal fluctuation, as changes in estrogen and progesterone levels or their depletion increase the risk of anxiety and depression in premenstrual syndrome, postpartum, and postmenopausal periods. TS can manifest with early ovarian failure, but the real contribution of hypogonadism regarding anxiety and depression disorders is yet to be established.^10,46^

The largest investigation to have been conducted into the mental health of young people with TS using standardized instruments, such as the Autism Diagnostic Interview, the Childhood Autism Rating Scale, and the Social Responsiveness Scale, found that compared to age-matched girls from the general population, they experienced higher rates of psychiatric and social skills difficulties. It was found that one-third of participants (34%) met criteria for a mental health disorder, a relative risk 2.6 times greater than typically developing girls.^47^

In the literature, ADHD has met diagnostic criteria in 13% of TS patients and ASD in 23% of participants, findings that agree with a Swedish study, which found an increased risk of clinically significant ASD in girls and women with TS. Considering relative risk (RR), it was 21.6 in ADHD and 57.5 in ASD compared to typically developing girls. Those who met ASD criteria also showed an increased risk of an associated emotional disorder (RR 2.9) and ADHD (RR 7.5) when compared with TS without ASD.^47^

A parental evaluation of TS patients using Social Responsiveness Scale-2 (SRS-2) found nearly two-thirds (61%) presenting autistic-like characteristics, which were considered to impact their day-to-day interactions, according to Wolstencroft et al.^47^ Considering both the DAWBA (Development and Well-being Behavior Assessment) and the SRS-2 parent’s analysis, approximately one in five girls met criteria for an ASD, which translates to a 57-fold RR of meeting ASD criteria in TS compared to girls from the general population.^47^ This notable finding is not found as strongly in other studies, although the risk for ASD is considerably higher, with some studies suggesting a fourfold greater risk of being diagnosed with ASD.^40^

It is advised that physicians who manage individuals with TS consider referring them for ASD assessment. Once the diagnosis is made, the patient should have social skills support and other therapies implemented — early intervention is the key to a good prognosis, such as gains in cognition, language, adaptive behavior, and improvements in daily living skills and social behavior.

It is hypothesized that the expression of autistic traits in TS could be influenced by epigenetic imprinting, such a point is supported by neuroimaging studies on brain development trajectories. TS individuals who inherited their single X from their mothers (~80%, Xm) present a more severe case of communication difficulties than those who inherited it from their fathers (Xp).

In the general population, the prevalence of epilepsy is slightly lower in women than in men. This small difference is not well described in the literature, so it is not possible to quantify it as a percentage.^48^

In TS, the neurological findings are often an integral part of the clinical symptoms. However, epilepsy and seizure reports are low. Also, the literature on them is rare, and the pathophysiological mechanism is still unclear. Research has shown the different encephalic gray matter volumes in X chromosome’s aneuploidies, including in TS.^49^

Raznahan et al. suggested a gradual increase in encephalic volume according to the number of X chromosomes present in the karyotypic constitution, which is called the “X chromosome dose-effect.”^50^ Therefore, the neuronal pathways involved may contribute to seizures and epilepsy findings.^51,52^ Furthermore, another explanation for epilepsy in TS could be the hormonal neuronal pathways, since sex hormone deficiency appears to influence seizure patterns.^53^

As it is a retrospective study, there are limitations regarding the number of patients that fulfill the inclusion criteria. For the same reason, the study lacks the use of specific tests that could quantify the neurophysiological abnormalities.

In conclusion, TS is the most frequent female SCA, although underdiagnosed. Our study showed that, despite being a widely known syndrome, there is still a knowledge gap that needs further research on the subject, especially regarding its association with neuropsychological findings. In addition, early diagnosis and treatment are essential to provide a better quality of life for these patients and, thus, contribute to social and economic improvements in society.

## Data Availability

The database that originated the article is available with the corresponding author.
